# Spatial Chromatin Organization Across the Cell Cycle: Insights from Auxin-Inducible Protein Depletion

**DOI:** 10.3390/cells15010051

**Published:** 2025-12-26

**Authors:** Artem Nurislamov, Anastasia Yunusova

**Affiliations:** 1 Scientific Center for Genetics and Life Sciences, Sirius University of Science and Technology, Sirius Federal Territory, Sochi 354340, Russia; 2 Institute of Cytology and Genetics, Novosibirsk 630090, Russia

**Keywords:** 3D genome, SMC complexes, CTCF, auxin-inducible degradation

## Abstract

Many cellular processes, including gene expression regulation, DNA replication and repair, as well as proper condensation and segregation of chromosomes, require highly coordinated remodeling of chromatin. Cohesin and condensins, the structural maintenance of chromosomes (SMC) protein complexes that function as ATP-powered loop extrusion motors, are key determinants of chromatin structure. The genetic loss of their function is lethal, whereas inducible degradation approaches enable rapid, robust analysis of the depletion phenotype. In this review, we discuss new insights into chromatin folding through each cell cycle phase from the auxin-inducible degradation (AID) system. We review the mechanisms by which condensins and cohesins contribute to the helical organization of mitotic chromosomes and to the maintenance of chromosome territories in interphase. Additionally, we discuss studies examining the roles of TOP2A, KIF4A, and SRBD during mitosis using the AID system. We then outline emerging principles of the mitotic-to-interphase transition and how targeted degradation of chromatin proteins reshapes this process. Finally, we highlight and discuss new advances in understanding interphase chromatin organization revealed by AID-based studies.

## 1. Auxin-Inducible Degron System

**Figure 1:**
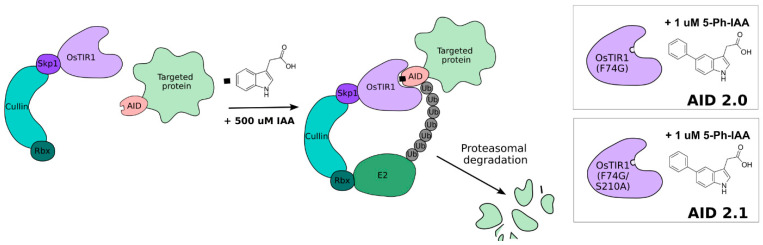
Scheme of the AID system. IAA binding to OsTIR1 induces the activity of the SCF/TIR1 complex recruiting an E2 ligase and promoting the ubiquitination and subsequent proteasomal degradation of the AID-tagged protein. Improved versions of OsTIR1 with a hole within the auxin-binding site are shown on the right of the Figure.

In eukaryotic cells, highly ordered chromatin structure plays an essential role in genome regulation and the underlying biological functions of chromosomes. Chromatin architecture is tightly coordinated in a spatiotemporal manner during cell cycle progression: from the relatively diffuse interphase chromatin structure, organized into dynamic DNA loops by cohesin and CTCF, to the highly condensed loop arrays of mitotic chromosomes mediated by condensins. The auxin-inducible degradation (AID) system, which allows the level of the target protein to be tightly controlled, has significantly advanced the knowledge of the key regulators of chromatin architecture and unveiled their novel functions. When combined with cell synchronization protocols, the AID system enables detailed analysis of proteins with stage-specific cellular function [1]. Briefly, this three-component system of protein degradation requires the following: (1) expressing an exogenous E3 ligase adaptor proteins (OsTIR1 or AtAFB2); (2) introducing an AID-tag, a short degron domain on N- or C-terminus of the target protein; and (3), adding the plant hormone auxin, indole-3-acetic acid (IAA), or its chemical analogs. IAA mediates the interaction between the E3 ubiquitin ligase SCF/TIR1 and a degron-fused protein, leading to its ubiquitination and proteasomal degradation (Figure 1). The AID system, as an effective approach for protein degradation, was pioneered in 2009, and for now it has been applied in many model organisms, including *Saccharomyces*, *Drosophila*, *Caenorhabditis*, zebrafish, mouse, chicken, and human cell lines, as has been thoroughly reviewed in [1,2,3].

The AID 2.0 system, based on the OsTIR1 (F74G) mutant, combined with the replacement of IAA by 5-Ph-IAA (with a 670-times lower concentration), allows for even more rapid protein degradation with reduced levels of basal degradation. Recently, an improved version of AID 2-43, AID 2.1, has been presented [4]. In this study, using base-editing-mediated mutagenesis for directed protein evolution, the authors generated OsTIR1 variants (S210A) that markedly enhanced the overall degron efficiency. The AID 2.1 system maintains high-efficiency protein degradation with minimal basal degradation and demonstrates faster target protein recovery after auxin withdrawal. Below, we discuss ongoing research using AID systems, which provides novel insights into the molecular mechanisms of mitotic and interphase chromosome folding.

## 2. Key Regulators of Mitotic Chromatin Architecture

The chromatin landscape is continuously remodeled throughout the cell cycle. Interphase chromosomes, which occupy defined regions within the nucleus, become highly compacted, forming the characteristic rod-shaped mitotic structures [5,6,7]. Such a high degree of compaction is influenced by both the loop extrusion activity of condensins and additional forces acting through extrusion-independent mechanisms [8,9]. Even after depletion of condensins, chromatin was still condensed, albeit no distinct rod-shaped chromosomes were observed [10]. Alterations in the molecular density around mitotic chromosomes after nuclear envelope breakdown also contribute to chromatin condensation [11]. In addition to condensins, several other factors play essential roles in establishing and maintaining mitotic chromatin folding, including cohesin, topoisomerase IIα, chromokinesin KIF4A, SRBD, and Ki67 [12,13,14,15].

### 2.1. Condensins

Two forms of eukaryotic condensins, condensin I and condensin II, belong to the Structural Maintenance of Chromosomes (SMC) protein family and share a similar architecture [16,17,18,19]. Each of them is composed of two core SMC subunits (SMC2 and SMC4) forming a V-shaped dimer with the hinge and two ATP-binding head domains; one kleisin subunit (NCAPH in condensin I and NCAPH2 in condensin II); and two non-kleisin subunits—HEAT-repeat–containing proteins (NCAPD2 and NCAPG for condensin I; NCAPD3 and NCAPG2 for condensin II).

Condensins have previously been shown to be molecular motors that actively extrude chromatin loops in an ATP-dependent manner [20,21]. Atomic force microscopy for single molecules of budding yeast condensin revealed that ATP binding induces a transition from open ‘O’ shape to collapsed ‘B’ shape, resulting in DNA loop extrusion by the scrunching mechanism [22,23]. Under such a mechanism, condensin stationary binds to DNA at the motor domain and transiently at the hinge domain. Upon ATP binding, a conformational transition to the B form causes the hinge domain to release and bind a new DNA. After multiple cycles of switching between O and B conformations, the size of the DNA loop significantly increases. Moreover, loop extrusion mediated by condensins induces positive DNA supercoiling [24,25]. Condensin preferentially binds to the tips of plectonemes and, by absorbing nearby plectonemes during extrusion, forms a supercoiled DNA loop. Such loops are highly stable and serve as focal points for the recruitment of additional condensin complexes [25].

Condensin activity is tightly regulated throughout the cell cycle to prevent premature chromosomal condensation. A variety of proteins, including MCPH1, M18BP1, and KIF4A, have been identified as regulators of condensin binding and loop extrusion [26,27,28,29,30]. In addition, condensin association with chromatin is modulated by cyclin-dependent kinases whose activities are restricted to particular stages of the cell cycle [19,31].

Condensin II is predominantly nuclear, which allows speculation about its role in interphase genome organization and its influence on genome function [32,33]. However, published data on this are somewhat inconclusive, and the extent of condensin’s effects is unclear [34,35]. In contrast, condensin I is cytoplasmic during interphase and binds chromatin only after nuclear envelope breakdown in prometaphase [36]. A fraction of condensin I remains in the newly formed nucleus after cytokinesis, but it is not chromatin-bound and is subsequently exported back to the cytoplasm [37].

**Figure 2:**
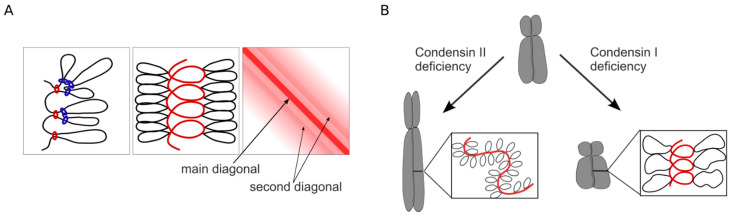
(**A**) Model of prometaphase chromosomes, where ~80-kb inner loops mediated by condensin I (blue rings) are nested within ~400-kb outer loops mediated by condensin II (red rings). The helical arrangement of consecutive loops (depicted as a red spiral) is visible on the Hi-C map as the second diagonal band. (**B**) Models of condensin I- and condensin II-depleted chromosomes. Depletion of condensin I leads to shorter and thicker chromosomes, while condensin II depletion makes chromosomes thinner and longer, preventing the helical winding of the scaffold.

The stoichiometry of condensin complexes determines the overall structure of mitotic chromosomes [38,39]. While condensin II mediates axial compaction, condensin I promotes lateral compaction; both of them are required for the mechanical rigidity of chromosomes to resist spindle forces during chromosome segregation [40]. Condensins can partially compensate for each other: in the absence of either condensin I or II, rod-like mitotic chromosomes can still form, albeit with some defects in condensation and segregation [19] (Figure 2B). At the same time, simultaneous loss of both condensins leads to more severe defects that completely abolish proper chromatid segregation, resulting in cell death [10].

Microscopy-based and Hi-C studies, combined with polymer modeling, suggested that condensin II forms long chromatin loops (up to ~500 kb) anchored to the chromosome scaffold. In contrast, condensin I generates smaller, nested loops (~80–100 kb) distributed more diffusely along mitotic chromosomes [41,42] (Figure 2A). The ratio of condensin II to condensin I is roughly 1:3–1:5 and varies across mitosis, reaching its most significant difference in anaphase [42]. Condensin II is stably bound to chromatin, while condensin I is more dynamic, presumably loading onto chromatin in multiple waves—first in prometaphase upon nuclear envelope breakdown and again in anaphase [42].

Condensin-mediated loops are not positioned at sequence-specific sites, in contrast to CTCF-mediated loops formed by cohesin. On Hi-C contact maps, condensin-driven loops appear as increased contact frequency near the main diagonal, reflecting local chromatin compaction rather than specific loop anchors [43] (Figure 2A).

#### 2.1.1. Condensins, Cohesin, and the Helical Structure of Mitotic Chromosomes

The question of how mitotic chromosomes are folded has long intrigued researchers, and with the development of novel techniques, continues to attract intense research efforts. Several models of mitotic chromosome organization have been proposed, which can be broadly divided into helical, non-helical, and semi-helical types—the latter suggesting that each chromatid is organized as a loop array along half-helical axes with alternating handedness [44].

Chromosome conformation capture (3C)-based data support the two-layered loop-extrusion model with a helically coiled scaffold, often referred to as a bottlebrush-like structure or a spiral staircase [41,45]. In such a structure, interactions between adjacent helical turns of chromatids give rise to structural periodicity, which appears on Hi-C maps as a “second diagonal” parallel to the main diagonal (Figure 2A). In synchronized DT40 CDK1a chicken cells, the second diagonal emerged in prometaphase and gradually shifted to larger genomic distances, from ~3 Mb to ~12 Mb, as cells progressed through mitosis. Using auxin-inducible degrons, it was shown that condensin II is required for establishing helical loop arrangements, as its depletion completely abolishes the second diagonal band [41,45]. It is worth emphasizing that condensin II loads onto chromatin during prophase, whereas the second diagonal emerges later in prometaphase. Apparently, its formation can also be due to other key scaffold proteins active at this stage, such as topoisomerase IIα and chromokinesin KIF4A. Interestingly, in several cell types, including mouse erythroblasts and neural stem cells, a weak second diagonal band is detectable even in interphase, suggesting the possible persistence of long-range contacts mediated by helical scaffold outside of mitosis [46,47]. The functional relevance of such contacts remains unclear.

In addition to condensin II, both condensin I and cohesin influence the helical structure of mitotic chromosomes [45]. Cohesin depletion with the AID system causes a drastic change in the position and prominence of the second diagonal band: it appears much more pronounced and is displaced toward larger genomic distances (from ~6 to ~8 Mb). This effect, clearly visible on Hi-C maps and the contact frequency p(s) plots, is supported by microscopy observations showing that cohesin-depleted chromosomes are wider and shorter [45]. Thus, cohesion of sister chromatids by cohesin partially restricts each chromatid’s ability to form helical arrays of condensed loops. After simultaneous loss of both condensin I and cohesin, Hi-C maps exhibit a strongly shifted second diagonal band up to ~16 Mb with additional third and fourth diagonals, corresponding to contacts between DNA regions separated by 2–3 helical turns [45]. This shift may indicate that, in the absence of condensin I and cohesin, each helical turn contains more DNA. It can be explained that solely acting condensin II either generates a higher number of loops per helical turn or extrudes chromatin into larger loops. Microscopy strongly suggests that condensin-II-only prometaphase chromosomes are even shorter and wider than cohesin-depleted chromosomes [45].

Helical arrangements of mitotic loop arrays across species and development. Several studies provided ample evidence for a clear relationship between the size of the inner and outer loops, DNA per helical turn, and chromosome size. In late prometaphase, condensed chicken chromosomes (0.6–196 Mb) are organized into 80-kb nested inner loops, arranged within a helical scaffold at 12 Mb per turn, with a height of one turn up to 200 nm [41]. In barley mitotic chromosomes, which are much larger (522–675 Mb), the second diagonal band is positioned at a larger distance, ~30 Mb, with a predicted helical turn of ~400 nm [48]. In axolotl mitotic chromosomes, which are gigantic in size (660–2960 Mb), the second diagonal band is also pronounced; DNA is folded into 600 Kb loops arranged into ~35 Mb helical turns, which are threefold longer than in chicken [49]. Thus, species with larger genomes require greater chromatin compaction, reflected in increased DNA per helical turn and a shorter helix. Analysis of contact frequency curves (P(s)) across different species (chicken, mouse, and human) showed that longer q-arms of chromosomes correspond to larger loops formed by condensins in mitosis [50].

It should be noted that mitotic chromosome length does not depend solely on genome size but also scales with cell and spindle size, as observed during early embryonic development, where rapid cleavage divisions drastically reduce cellular and nuclear volume [51]. Experiments using Xenopus egg and embryo extracts have shown that the reduction in chromosome size during development results from decreased condensin I loading onto chromatin, leading to an increase in the amount of DNA per turn [52]. This demonstrates that modulating condensin stoichiometry provides a robust means to regulate mitotic chromosome folding at species- or stage-specific levels.

#### 2.1.2. Condensins, Cohesin and the Transition of Mitotic Architectural Features in the Subsequent Interphase

**Figure 3:**
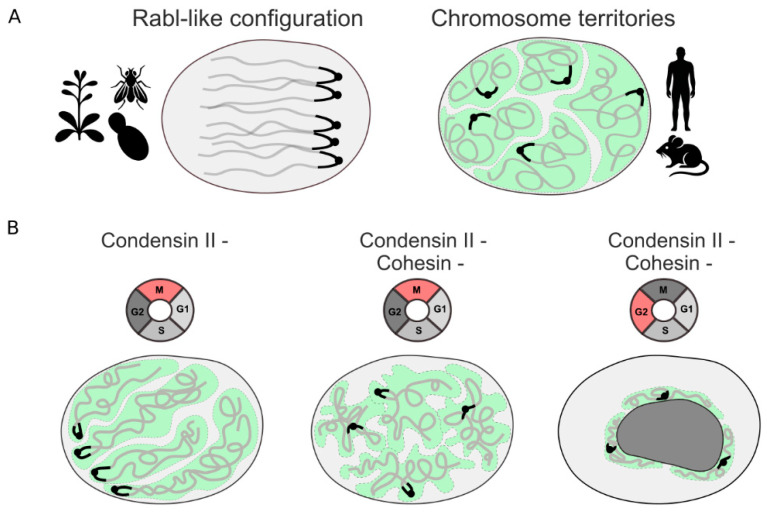
(**A**) Patterns of chromosome arrangements in interphase: Rabl-like configuration with centromeres and telomeres clustered at the opposite sites of a nucleus and individual chromosome territories (CT) that occupy distinct positions within the nucleus. (**B**) The structures of chromosome territories after depletion of condensin II, solely or combined with cohesin depletion, through mitosis and during G2. In the absence of condensin II during mitosis and transition to G1, CT adopted an elongated structure with hyper-clustering of centromeric and pericentromeric heterochromatin. When both condensin II and cohesin are depleted during mitosis, CTs are expanded and transformed into cloud-like areas with no centromeric clustering. Double depletion of both complexes in G2 leads to the collapse of CT and chromosome redistribution around the nucleolar periphery.

At the scale of whole chromosomes, several types of chromatin configurations have been described [53]. Broadly, they can be divided into two major categories. The first type corresponds to chromosome territories (CT), in which each chromosome occupies a discrete territory within the nucleus [54] (Figure 3A). The second type is the Rabl configuration, characterized by clustering of centromeres and telomeres at opposite poles of the nucleus [55] (Figure 3A). An intermediate type can be defined as rosette-like, exemplified by *Arabidopsis thaliana*, whose chromosomes form distinct territories with densely packed centromeric heterochromatin (chromocenters), while telomeres cluster around the nucleolus [56]. CT-like configurations are typical for mammals, whereas Rabl-type configurations are more common in plants, mosquitoes, and flies. Interestingly, a preprint study reported that the Rabl-like configuration may not be species-specific but rather a developmental stage [57]. Thus, Rabl-like chromosome polarization was observed during early development of mouse embryos from the 2- to 8-cell stage. Chromatin configuration switching occurred until the 64-cell stage, when discrete CTs were almost established [57].

Increasing evidence indicates that the mitotic chromosome folding, and specifically the stoichiometry of condensins and cohesin, affects the spatial distribution of chromosomes in the subsequent interphase [58]. Comparative analyses of 3D genomics across species have revealed a correlation between the loss of condensin II and the Rabl-type configuration [59]. Indeed, in *Anopheles* mosquitoes, *Drosophila melanogaster*, *Saccharomyces cerevisiae*, and some other species with pronounced Rabl-like architecture, one or more subunits of the condensin II complex are lacking [59]. Because these organisms are evolutionarily distant, it is evident that the loss of condensin II components has occurred independently multiple times, suggesting that switching between chromatin configurations may have functional significance.

Experimental depletion of condensin II supports the inverse correlation between condensin II activity and Rabl-type configuration. In both mammalian and plant cells, loss of condensin II during mitosis leads to centromere hyperclustering and the formation of large heterochromatic chromocenters in the subsequent interphase, which are very reminiscent of the Rabl-like architecture (Figure 3B) [59,60,61]. Such rearrangements may result from retaining memory of the anaphase folding with centromere adhesion and a parallel arrangement of chromosome arms [62]. Presumably, impaired axial shortening followed by increased chromosome intermingling hinders proper chromosome redistribution in the newly formed nuclei [59]. Remarkably, this large-scale reorganization has minimal impact on gene expression. Only a small subset of genes, mainly located within or near Lamina-Associated Domains (LADs), alter their activity after condensin II depletion, likely reflecting changes in centromere repositioning [59,60,63].

An interesting observation was made in a study of the chromatin architecture of motor neurons during maturation [64]. It turned out that even in post-mitotic, non-dividing neurons, chromatin reorganization occurs, accompanied by an increase in the frequency of telomere–telomere and centromere–centromere contacts. This suggests that the Rabl-like configuration can be actively established in the interphase nucleus under the influence of undetermined forces and is probably not simply a consequence of the inheritance of chromatin folding during anaphase of mitosis.

Orthogonal multiscale fluorescence in situ hybridization (FISH) studies using AID-mediated degradation of condensin II and cohesin have refined and extended the role of these SMC complexes in establishing and maintaining CT (Figure 3B) [65]. According to previous data, cohesin depletion alone had little effect on CT morphology, whereas condensin II loss caused CTs to lose their spherical shape and become elongated, accompanied by centromere clustering. Simultaneous depletion of cohesin and condensin II produces diffuse, cloud-like CT occupying a larger nuclear volume, without distinct centromere clustering. This suggests that the contribution of cohesin to CT organization was previously underestimated, as its effect is normally masked by condensin II activity. Even more intriguing are the results from G2-phase cells showing that simultaneous depletion of cohesin and condensin II during interphase leads to drastic changes in chromosome configuration. CTs acquire an abnormally compact morphology, predominantly localized around the nucleolar periphery, resembling the “surrounded nucleolus” configuration seen in mouse oocytes and two-cell-stage embryos [66,67]. Because nuclei at these developmental stages are substantially larger than those of cultured cells, the authors proposed that these specific configurations may arise from SMC complex depletion mechanisms, reflecting condensin and cohesin exhaustion [65]. These data collectively support a cooperative role for cohesin and condensin II in maintaining interphase chromatin configuration.

Interestingly, depletion of condensin I during mitosis does not alter chromosome configuration [59]. Moreover, loss of condensin I components during evolution is much rarer. Notable examples include tardigrades, which lack condensins entirely, and Arctic lampreys, which have lost the non-kleisin subunits of condensin I (Cap-G and Cap-D2) [59]. This suggests that condensin I is more critical for cell viability, raising the question of how such organisms tolerate condensin I loss during cell division.

Taken together, it appears increasingly likely that mitotic chromosome folding, in conjunction with interphase processes such as cohesin-mediated DNA loop extrusion, determines the configuration of interphase chromosomes. Experimental data demonstrate that disruption of mitotic folding; for example, reduced axial compaction in the absence of condensin II leads to centromere clustering and a shift from CT to a Rabl-like configuration.

#### 2.1.3. Condensins and Cohesin: Interaction of Loop Extruders During the Cell Cycle

Cohesin, the primary loop-extruding complex in interphase, is replaced in prophase by condensin II. Using chicken DT40 cells synchronously progressing through mitosis, combined with the AID system, it was examined how these SMC proteins guide mitotic folding, and the “Rules of engagement for condensins and cohesins” were formulated [45].

First, condensins can bypass cohesin complexes. The combined analysis of microscopy and simulation revealed that condensins can pass through cohesive cohesin that connects sister chromatids, effectively traversing the entire chromatid [45].

Second, condensins promote removal of extrusive cohesin from chromatin. Normally, interphase features, such as (topologically associating domains) TADs (topologically associating domains), loops, and stripes, visible on Hi-C maps, disappear in prometaphase. However, upon acute condensin depletion, these structures persist longer, implying a role for condensins in displacing cohesin-mediated loops during mitotic entry. Condensins can facilitate the displacement of cohesin either by pushing it along the chromatin fiber or by promoting its unloading. The latter mechanism is considered more plausible, as condensins are relatively weak motors and unlikely to exert strong mechanical pushing forces [68,69]. Condensins are not the only players; additional background processes likely contribute, such as CTCF unloading. Interestingly, Mcph1 knockout, which causes premature loading of condensin II in interphase, induces early chromosome condensation during G1/G2 phases but does not disrupt cohesin-dependent chromatin loops [28]. Assuming condensin II remains extrusion-competent after Mcph1 knockout, this suggests that the interplay between cohesin and condensins may be cell-cycle specific, functioning differently between mitosis and interphase.

Third, condensins stall upon collision. Condensin II complexes appear to halt upon encountering each other. Supporting this, condensin-mediated loops expand to ~400 kb by prophase but cease to grow thereafter, coinciding with the establishment of a chromosome scaffold enriched in condensin within the chromatid interior. It is also supposed that longer chromatin loops correlate with larger spacing between condensin complexes [48]. In polymer simulations of chicken macrochromosomes, the inter-condensin distance was set at ~10 nm, whereas analogous modeling of barley mitotic chromosomes predicts a larger spacing of ~100 nm with loop-free chromatin regions [48]. This may be explained by the fact that longer loops impose topological constraints that can impede extrusion by neighboring condensins, consistent with their low opposing force and weak motor properties [70]. However, in barley, condensin II complexes do not accumulate along the central chromosome axis, but instead display a dispersed distribution along chromosome arms, with enrichment in centromeric regions [48]. Presumably, other factors, such as topoisomerase IIα, modulate condensin spacing by influencing supercoiling and loop-relaxation dynamics.

It should be noted that this “collision-and-stalling” model is still debatable. Alternative models propose that condensins can traverse one another rather than stop upon contact. For example, according to an imaging–based simulation study with nanoscale resolution [71], condensin II was proposed to pass through neighboring complexes, thereby forming large, overlapping loops reaching 6–8 Mb in length. Another similar model introduces the concept of “super-extruders”, representing a third hierarchical level of chromatin folding [72]. In this model, the bottlebrush-like scaffold generated by condensins undergoes axial compression due to the action of very large loops (up to 10 Mb) formed by super-extruders. Super-extruders are hypothesized to pass through condensin complexes, with 20–200 such extruders per chromosome sufficient to achieve the desired mitotic chromatin folding. Moreover, polymer simulations predict spontaneous helical ordering, manifesting as a second diagonal in Hi-C contact maps [72]. Since the second diagonal disappears upon condensin II depletion, condensin II emerges as the prime candidate for the role of a super-extruder driving large-scale helical folding of mitotic chromosomes.

### 2.2. Topoisomerase IIα

The type II topoisomerases function as dimers that alter DNA topology by introducing transient double-stranded breaks in one DNA duplex and transporting another DNA duplex through the gaps [73,74]. Topoisomerase IIα (TOP2A), the most abundant protein in the scaffold, exhibits both catenation/decatenation activity through mitosis [12,75]. First, TOP2A resolves inter-chromatid entanglements to ensure individualization, and then promotes intra-chromatid entanglements, maintaining the stiffness of mitotic chromosomes and leading to chromatid thickening [76,77]. Moreover, TOP2A performs sufficient resolution of sister chromatid interwines during anaphase and regulates disentanglement during mitotic exit, both of which are required to establish a normal architecture of territorial interphase chromosomes [78,79].

The functional transition of TOP2A from catenation to decatenation activity is modulated by additional mechanical forces exerted on chromatin. For instance, the loop extrusion activity of condensins introduces positive DNA supercoiling, thereby generating tension on sister chromatid intertwines, which, in turn, stimulates TOP2A activity for decatenation [80,81].

A study using the AID system reported that TOP2A is not involved in prophase but is particularly important in prometaphase, as its depletion impairs chromosome individualization and almost completely abolishes chromatid segregation [82]. Importantly, acute depletion of TOP2A during prometaphase or metaphase leads to decondensation of already condensed chromosomes. These results suggest that condensins establish the overall shape of the mitotic chromosome, whereas TOP2A locks and maintains that structure once formed. Inhibitor studies further support this structural maintenance role: blocking the catalytic activity of TOP2A with etoposide or ICRF-193, which immobilizes TOP2A on chromatin, does not disrupt chromosome condensation in pre-condensed prometaphase cells [82].

Furthermore, depletion of TOP2A does not affect the localization pattern of other scaffold proteins such as condensins or KIF4A. However, a modest irregularity in the distribution of condensin II has been observed [82]. Such a change is often accompanied by long and thin chromosomes with helically twisted scaffolds that phenocopy the effects of condensin II depletion [13,82]. Taken together, these findings led to a conceptual model in which TOP2A acts as a molecular clamp that embraces condensin-generated loops, thereby bringing them into proximity and contributing to axial shortening of chromosomes [82].

### 2.3. Chromokinesin KIF4A

KIF4A is a dimeric plus-end-directed motor protein associated with microtubules. It localizes to the nucleus during interphase and redistributes to the mitotic spindle and midbody during mitosis [83]. Along with condensins and TOP2A, KIF4A is a core component of the mitotic chromosome scaffold, contributing to its structural integrity [13]. KIF4A is dynamically associated with chromatin and is colocalized with condensin I in a punctate pattern along the chromosome axis, alternating with TOP2A [13].

Recent work has demonstrated that KIF4A directly interacts with the NCAPG subunit of condensin I, competing with the NCAPH and NCAPD2 [29]. The latter two subunits maintain condensin I in an autoinhibited conformation that is analogous to MCPH1 regulation of condensin II. The binding of KIF4A induces conformational changes that allow DNA threading through the NCAPH kleisin, initiating loop extrusion [29]. Loss of KIF4A reduces condensin I loading onto chromatin and phenocopies condensin I depletion in regulating lateral chromosome compaction [13]. Furthermore, the regulation of the KIF4A–condensin I interaction involves kinase signaling: inhibition of Aurora B kinase weakens condensin I/KIF4A chromatin binding [84].

The depletion of KIF4A with auxin resulted in defective Chromosomal Passenger Complex (CPC) localization to the spindle midzone during anaphase [85]. Uniquely among kinesins, KIF4A possesses nuclear localization and direct DNA-binding capacity [86]. *Kif4a* knockout in mouse ES cells results in abnormal chromatin reorganization, specifically and predominantly affecting heterochromatic regions, leading to their decondensation and transcriptional activation. This effect may be linked to the interaction between KIF4A and Poly(ADP-ribose) Polymerase 1 (PARP-1). Loss of KIF4A results in a marked increase in ADP-ribosylation of core and linker histones, leading to a more open, transcriptionally active chromatin state [86].

### 2.4. S1-Domain–Containing Protein 1 (SRBD1)

Recently published studies have described a novel role for SRBD1 (S1 domain-containing protein 1) [15,87]. Using genetic screens, SRBD1 has been identified as a key and evolutionarily conserved component of the mitotic chromosome scaffold. SRBD1 contains RNA- and DNA-binding domains with low sequence specificity [88]. Interphase cells show prominent nuclear localization of SRBD1 with its highest abundance in the nucleolus. Then, during prophase, it is recruited to chromatin and remains bound throughout mitosis, showing irregular axial localization enriched in centromeric regions. Using the AID system for targeted degradation, these two independent studies demonstrated that SRBD1 is crucial before prometaphase, since its depletion during anaphase or later does not cause severe phenotypes or lethality [15,87]. The effects of SRBD1 loss closely resemble TOP2A inhibition, including defective axial shortening, chromatid intertwining, anaphase bridges, micronuclei formation, and cell death. However, results regarding the relationship between SRBD1 and TOP2A are ambiguous. In a study by Lovejoy et al. using HCT116 cells, it was shown that SRBD1 depletion disrupts TOP2A localization [15]. In contrast, Raaijmakers et al. reported that in HAP1 cells, SRBD1 loss does not TOP2A activity or distribution [87]. Both studies, however, agree that co-depletion of condensin II partially rescues the SRBD1 depletion phenotype, although the mechanism remains unclear. It has been hypothesized that SRBD1 likely participates in prophase, when chromosome condensation is already established, helping to resolve catenane structures induced by condensin II, TOP2A, or RNA polymerases [87].

### 2.5. Antigen Kiel 67 (Ki67)

Ki-67 is a well-established marker of cell proliferation, often used in cancer diagnostics [89,90]. Being a nucleolar protein, during mitosis, Ki67 localizes to the surface of chromosomes, forming a distinct perichromosomal layer [91]. Due to its surfactant properties, Ki-67 acts as a sheath that promotes individualization of chromosomes and prevents clumping, thereby ensuring proper spatial segregation of sister chromatids during mitotic exit [92]. Additionally, Ki-67 contributes to the organization and equal redistribution of nucleolar components into daughter cells [93]. Despite its diagnostic importance, the molecular functions of Ki-67 have remained elusive, mainly because of challenges in disentangling its cell-cycle-dependent roles and compensatory mechanisms in long-term knockdowns or knockouts [94,95]. Compared with previous studies, the AID system has allowed for rapid depletion of Ki67, enabling precise analysis of its function during specific cell cycle stages [96,97,98]. Thus, depletion of Ki67 specifically after mitotic entry resulted in structural disorganization of preassembled rod-shaped chromosomes, which became swollen and distorted [98]. Moreover, the central axis localization of TOP2AIIα and condensin II was disrupted upon Ki-67 removal, suggesting an interplay between chromosome structure proteins and perichromosomal layer [98]. Further experiments demonstrated that simultaneous depletion of Ki-67 and condensin II resulted in more severe morphological defects (ball-like chromosome clusters) distinct from those observed with depletion of either factor alone [97]. These findings suggest that condensin II and Ki-67 contribute independently but cooperatively to maintain mitotic chromosome architecture.

Beyond its mitotic functions, acute depletion of Ki-67 at the G1/S boundary revealed a novel role during DNA replication [96]. Removal of Ki-67 at this stage led to severely delayed S phase, unloading the replication machinery and causing massive DNA damage that was not sensed by canonical DNA damage response pathways [96]. Additionally, sister chromatid cohesion in G2 phase was also impaired following Ki67 depletion at the beginning of S phase [96]. Presumably, it can be explained by WAPL-mediated cohesin removal triggered by replication stress [99].

## 3. New Insights into Post-Mitotic Chromatin Reorganization

Upon exit from mitosis, chromatin undergoes drastic topological changes to establish interphase chromatin conformation. In most eukaryotic species, interphase chromatin is organized into spatially separated structures-TADs. Vertebrate TAD formation is primarily mediated by a cohesin/CTCF-dependent mechanism [100,101,102,103,104], in which the cohesin complex extrudes chromatin loops with frequent direction switching [105] until encountering CTCF-bound sites, where extrusion stalls through cohesin interactions with the CTCF N-terminal region [106,107,108,109,110]. Such a difference from mitotic chromatin organization requires a transition from condensin-packed chromosomes to interphase architecture with domains and compartments. This transition enables daughter cells to re-establish chromatin architecture, reconstitute cell-type-specific regulatory patterns, and recover the transcriptional program of the parent cell.

**Figure 4:**
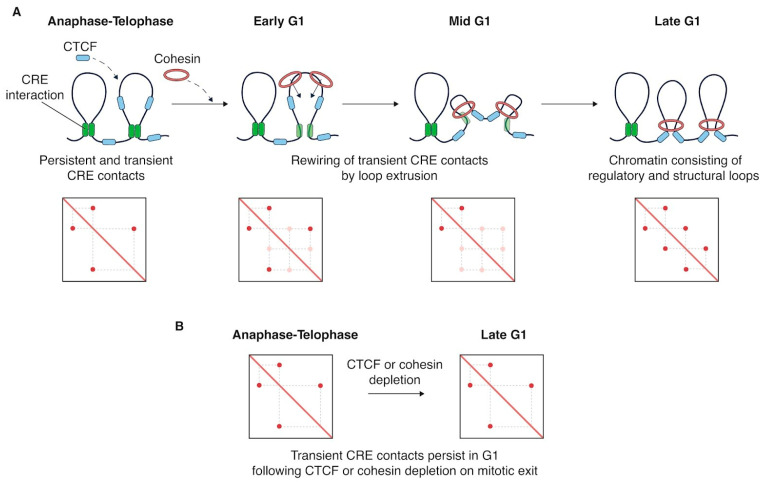
Reorganization of chromatin loops during mitotic exit. (**A**) Model illustrating sequential reorganization of chromatin loops from anaphase-telophase to late G1. CRE-anchored contacts intensify in anaphase-telophase independently of CTCF (rebinds chromatin in anaphase) and cohesin (rebinds chromatin in late telophase after nuclear envelope reformation). During G1 re-entry, loop extrusion machinery rewires transient contacts by forming structural chromatin loops. By late G1, chromatin architecture is restored with both structural and regulatory loops. Below, the Hi-C map contact pattern represents changes in loop organization on mitotic exit. (**B**) Transient CRE contacts that form during mitotic exit persist in G1 upon CTCF or cohesin depletion, showing that CRE-driven interactions can arise independently of cohesin-mediated extrusion.

Interestingly, interactions between cis-regulatory elements (CREs) are detectable from prometaphase onward, even though transcription is silent at this stage [111,112,113]. These contacts are also observed in telophase (Figure 4A), when condensins have already disengaged from chromatin while cohesins have not yet initiated loop extrusion [114]. CRE-anchored interactions are likewise present in condensin-depleted mitotic chromatin [115]. Together, these observations indicate that such contacts can persist transiently or reform independently of canonical loop-extruding complexes. As mitosis proceeds, these focal CRE interactions become more pronounced, promoting the emergence of CRE-enriched microcompartments, which subsequently diminish upon G1 re-entry [113]. The re-establishment of CRE contacts upon G1 re-entry can be facilitated by the inherent ability of chromatin with similar biochemical properties to self-aggregate. These affinity-driven associations between regulatory elements could reflect a form of mitotic bookmarking. This chromosome-intrinsic folding program presumably facilitates rapid re-establishment of regulatory interactions upon mitotic exit [116].

Depletion of the cohesin loader NIPBL during the M–G1 transition [117], or preventing cohesin nuclear import via RanGAP1 or Nup93 depletion [114], weakens structural loops in G1 cells and has a stronger impact on transcription than cohesin depletion in asynchronous cells. Although most structural loops are lost, CRE-anchored interactions persist in G1, indicating that cohesin is primarily required for forming structural loops after mitosis. In contrast, regulatory loops can assemble independently of cohesin-mediated extrusion [118]. Interestingly, transient CRE-anchored loops and microcompartments, which normally weaken or disappear upon G1 re-entry [111,112,113], persist when cohesin loading is prevented at mitotic exit [114,117], suggesting that loop extrusion rewires these transient interactions during interphase entry.

In contrast to cohesin, CTCF rebinds chromatin earlier, during anaphase [111,119], before formation of nuclear envelope [114]. Acute CTCF loss during G1 re-entry disrupts structural loops and expands loop size, consistent with its role in delimiting topological boundaries. Similarly to cohesin depletion, transient CRE-anchored loops that normally disappear after telophase persist upon CTCF loss, suggesting competition between regulatory (CRE-anchored) and structural (CTCF/cohesin-dependent) interactions (Figure 4B) [120]. Early CTCF rebinding appears to demarcate future domain boundaries that are later established by loop extrusion.

Disruption of either factor specifically during mitotic exit has a more pronounced impact on chromatin topology than depletion during interphase [117,118,120,121]. This reflects a dual role of CTCF and cohesin in mitotic exit: rewiring transient contacts [113,114,118,120] and establishing the structural framework for CRE interactions that form in G1 [122]. Moreover, loop extrusion facilitates reformation of loops with weak enhancers [118]. Higher-order structures such as TADs, which also rely on cohesin/CTCF-mediated loop extrusion, reappear in a bottom-up manner during G1 re-entry: subTADs form first, followed by the assembly of TADs composed of multiple subTADs [111,112]. Together, these observations outline a temporal hierarchy of postmitotic chromatin reorganization that ensures the rapid restoration of genome architecture after cell division.

Mitotic exit is also accompanied by a wave of transcriptional reactivation [123,124]. Furthermore, CRE-driven loops and microcompartments that emerge throughout mitosis are associated with transcriptional activity at the M-G1 transition [113], suggesting a role for CRE-driven structures in transcriptional reactivation upon mitotic exit. Since regulatory loops and microcompartments are present throughout mitosis [113], this indicates that their formation does not require transcription. Indeed, RNA Polymerase II (RNAPII) binds chromatin only after nuclear envelope reformation [114], which also highlights the contribution of inherited cytoplasmic factors to interphase chromatin reassembly in addition to chromosome-intrinsic factors. Auxin-induced degradation of RPB1, the largest subunit of RNAPII, is commonly used to study RNAPII depletion [125,126,127,128,129], as it is essential for polymerase function. Acute loss of RNAPII on mitotic exit reduces chromatin-associated NIPBL levels, possibly due to decreased cohesin recruitment at actively transcribed regions. Conversely, RNAPII loss unexpectedly increases chromatin accessibility, suggesting that RNAPII may act as a moving barrier or an anchor for cohesin, thereby helping define loop boundaries. As a consequence, upon G1 re-entry, TADs become markedly weakened, accompanied by compartment mixing and an increase in interchromosomal contacts [128,130]. Thus, RNAPII-driven interactions are crucial for the formation of interphase chromatin after mitosis and for linking transcriptional activity to subsequent cohesin loading. Notably, the impact of RNAPII depletion on chromatin topology is far more pronounced during G1 re-entry than in asynchronous interphase cells, where topological changes are confined mainly to altered looping patterns [128].

## 4. Dissecting Interphase Chromatin Architecture Through Degron Systems

**Figure 5:**
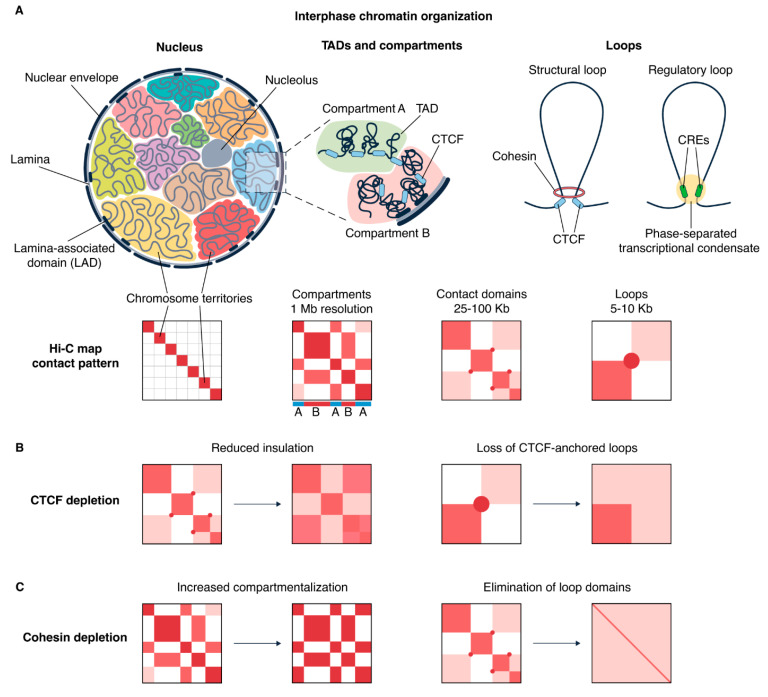
Hierarchical organization of interphase chromatin. (**A**) Schematic overview of interphase chromatin architecture and its representation on Hi-C contact maps at different scales. Chromatin is organized into chromosome territories, A/B compartments, TADs, and loops, with distinct structural (cohesin/CTCF-dependent) and regulatory (CRE-anchored) configurations. (**B**) Acute loss of CTCF during interphase reduces insulation and disrupts CTCF-anchored loops. (**C**) Cohesin depletion enhances compartmentalization and eliminates loop domains.

Interphase chromatin is organized into a multi-layered, hierarchical structure (Figure 5A) that supports tissue-specific transcriptional programs. In addition to CTCF and cohesin, which contribute to the formation of vertebrate TADs [102,103,104], the folding of interphase chromatin at the level of loops and domains can also be mediated by other mechanisms. The transcription machinery also contributes to shaping chromatin architecture by generating negative supercoiling [131,132] and acting as a semipermissive moving barrier for cohesin-mediated loop expansion [133,134,135]. Cohesin-driven looping itself can also induce negative DNA supercoiling during extrusion [136]. Supercoiled regions are regulated by topoisomerases, which accumulate at loop bases and resolve topological stress induced by supercoiling [137,138]. This molecular interplay highlights the complexity of the mechanisms that actively shape chromatin architecture. Here, we summarize and discuss the main discoveries in interphase chromatin organization revealed through auxin-inducible degradation systems.

### 4.1. Interphase Chromatin in the Absence of CTCF

Since CTCF is essential for cell survival in vitro and during early development, as well as core loop extrusion machinery subunits, loss-of-function studies of architectural chromatin proteins have historically faced significant challenges [139]. In cell culture, CTCF knockout reduces proliferation and triggers cell death [140,141,142], whereas in mice, knockout or knockdown is lethal at the blastocyst stage [143]. Similarly, in Drosophila, CTCF knockout is lethal during the larva-to-pupa transition [144]. Conditional knockout provides an alternative approach to study CTCF loss-of-function effects in both cell culture and in vivo. Importantly, this method overcomes the developmental lethality observed in conventional knockouts and allows tissue-specific functional studies [145,146,147]. However, conditional knockouts are not reversible, as recombinase permanently removes the floxed site from the genome, preventing investigation of protein recovery effects. Also, Cre-mediated recombination occurs asynchronously across cells and may take days to deplete protein levels fully. Additional technical challenges may arise with tamoxifen-inducible systems, as tamoxifen and Cre-ERT2 have been reported to exhibit toxic effects in mice and in cell culture [148,149,150,151,152]. RNAi is another indirect method of protein knockdown, since siRNA/shRNA targets mRNA rather than the protein itself [153]. Thus, it can take days for protein depletion, allowing cells to undergo multiple divisions with gradually decreasing protein levels, leading to possible accumulation of secondary effects [154]. The knockdown of chromatin proteins by RNAi is often incomplete—for example, some CTCF ChIP-seq peaks remained detectable [155], while TAD disruption appeared limited following RAD21 knockdown [156].

The emergence of the AID system has overcome the experimental disadvantages of previously described methods and greatly expanded current knowledge of CTCF functions. As a result, targeted CTCF degradation has revealed multiple effects on chromatin structure and transcriptional activity. CTCF depletion in human and mouse cells leads to genome-wide disruption of loops and significantly reduced TAD insulation [157,158,159,160], underscoring its architectural role (Figure 5B). While disrupting loop anchors and reducing TAD insulation, CTCF depletion did not affect compartmentalization, suggesting its limited role in higher-order chromatin organization. Washing off auxin restores protein levels and chromatin occupancy, thereby rescuing its functions [157].

While CTCF serves both architectural and transcriptional roles [161,162], its depletion shows surprisingly modest effects on gene expression—short-term auxin exposure, sufficient for loop disruption and reduced TAD insulation in asynchronous cells, alters only a few hundred genes [157,159,160]. Long-term depletion, however, leads to more pronounced changes in gene expression [107,163]. Consistent with these observations, short-term CTCF degradation was shown to have only minor effects on promoter–enhancer interactions [163,164,165]. Is CTCF truly dispensable for establishing promoter–enhancer interactions? Not quite. More likely, CTCF contributes to the initial formation of these loops. Still, promoter–enhancer contacts can persist following CTCF depletion with only modest transcriptional perturbations, possibly due to phase-separated condensates formed by transcriptional coactivators, which may help maintain these interactions in the short term [122]. Additionally, in the absence of CTCF, promoters were shown to rewire their interactions from distal to proximal enhancers to sustain their activity, demonstrating robustness to CTCF depletion [163,165]. Thus, transient stability in promoter–enhancer interactions may safeguard transcriptional programs against cellular perturbations. In summary, cohesin/CTCF loops are essential for establishing structural loops during post-mitotic chromatin reorganization and for defining cell fate during differentiation [142,163], but are mostly dispensable for their long-term maintenance [166].

### 4.2. Exploring CTCF’s Functions Through Complementation Assays

The AID system provides a versatile platform not only for probing loss-of-function phenotypes but also for complementation approaches. Following degradation of the AID-tagged endogenous protein, transgenic variants can be introduced to rescue the phenotype or test for specific mutations. This strategy was successfully employed to identify cohesin-interacting motifs in the CTCF N-terminal region [107] that allow CTCF to block cohesin translocation and protect cohesin from unloading by WAPL, thereby explaining why cohesin stalls at CTCF sites in an orientation-specific manner. Mutations in the key cohesin-interacting motif YDF produced an effect similar (though not completely) to endogenous CTCF depletion, highlighting this motif’s essential role in cohesin-CTCF interactions during chromatin loop formation [106,107,110].

CTCF is an established tumor suppressor [167,168] that is frequently mutated in endometrial cancer [169] and other types of tumors. Functional characterization of these mutations is therefore critical for understanding the molecular mechanisms driving oncogenesis. The initial strategy for CTCF cancer mutation screening was employed in K562 cells [170] by introducing HA-tagged CTCF mutant variants via lentiviral transduction to distinguish transgenic CTCF from endogenous CTCF. This approach revealed mutation-specific effects on proliferation and binding, with potential caveats, including competition between endogenous and mutant CTCF and increased protein dosage due to coexpression. The complementation system overcomes these limitations, as demonstrated by Do et al. [171], who investigated how cancer-associated mutations in CTCF’s zinc fingers (ZF) alter its binding profile, chromatin accessibility, residence time, and gene expression in mESCs. The differentially expressed genes identified across cells with expressed mutant variants showed enrichment for cancer- and immune-related processes, consistent with the disease associations of the studied mutations. These findings showcase how complementation assays can enable functional analysis of cancer-associated variants. Furthermore, this approach could be extended to investigate N-terminal mutations (e.g., Y226C [170,172]), which may disrupt interactions with cohesin during loop formation.

Beyond its canonical DNA-binding and cohesin-related functions, CTCF has been reported to bind RNA [173,174], and complementation assays have been widely used to assess the role of putative RNA-binding regions. Notably, expression of CTCF variants carrying mutations in these regions leads to a pronounced, genome-wide disruption of chromatin loops [174,175], comparable to phenotypes observed for cohesin-interacting and DNA-binding mutations. However, as the RNA-binding activity of many chromatin-associated proteins remains debated [176,177], it is unclear whether these effects directly reflect RNA-mediated interactions or arise from altered DNA binding or protein structure. Consistent with a potential regulatory role, RNA-dependent modulation of CTCF insulation has been reported at the INK4a/ARF and MYC TAD boundaries, where enhancer-driven transcription strengthens CTCF occupancy and domain insulation [178]. Thus, whether and how RNA contributes to CTCF chromatin binding and function remain open questions.

Complementation assays provide a robust framework for investigating domain-specific protein functions and cancer-associated mutations, as demonstrated by studies of CTCF. More broadly, this strategy can be extended to probe evolutionary changes in loop extrusion machinery, chromatin remodelers, and other chromatin-associated complexes in cell culture. Below, we outline several auxin-mediated complementation strategies and compare their advantages and limitations.

An approach relying on the random integration of both OsTIR1 (AID1) and a transgene encoding CTCF variants, the latter introduced via lentiviral transduction [174], provides a straightforward method for complementation assays. Limitations arise from the use of constitutive expression, which restricts temporal control of transgene expression. Additionally, this method requires cell sorting to isolate transgene-positive cells, though, in theory, sorting could be replaced with alternative strategies, such as puromycin selection. An improved approach was employed by Hyle et al. [179,180], which used lentiviral integration of both OsTIR1 (AID2) and transgenes with antibiotic selection. Transgene expression was also controlled by a doxycycline-inducible promoter, enabling tunable expression conditions. Overall, the latter approach offers controllable transgene expression conditions, reduced basal degradation, and more efficient depletion of AID-tagged protein

Even though safe harbor homozygous integration with the Tet-On promoter provides controllable, uniform transgene expression across cells, transgene expression levels were lower than those of endogenous AID-tagged CTCF [107,171]. Nevertheless, wild-type transgene expression levels were sufficient to rescue protein binding to 97% of CTCF-motif-containing sites. Despite requiring extensive work to generate homozygous knock-ins, this approach offers a viable, highly controllable option. Combined with novel AID versions to reduce basal degradation, this experimental design could be further improved [175]. However, the lower transgene expression from the Tet-On promoter may pose a problem when complementing proteins with high endogenous expression levels, making viral integration at a high multiplicity of infection (MOI) a potentially more reliable alternative in such cases.

The most straightforward approach for a complementation system could use an integration-free plasmid strategy. While offering a less laborious option, plasmid complementation would suffer from non-uniform transient overexpression, as dozens or even hundreds of plasmids can enter the nucleus after transfection [181]. As shown for RAD21, overexpression can introduce significant artifacts: excessive cohesin levels lead to vermicelli-like chromatin structures due to accumulated cohesin bound to chromatin, resembling the effect of WAPL depletion [182]. Overexpression can also lead to non-specific chromatin binding, reducing the reliability of downstream analyses.

Regardless of OsTIR1 and transgene integration methods, we suggest using AID 2.0 [183] or AID 2.1 [4] systems for depletion and complementation assays, as improved AID versions offer rapid protein depletion and less leaky degradation in the absence of ligand.

### 4.3. Cohesin and Its Cofactors: Effects on Interphase Chromatin Topology and Gene Expression upon Depletion

Targeted degradation of core cohesin subunits disrupts chromatin domain integrity and reduces insulation [158,184], underscoring cohesin’s essential role in interphase chromatin maintenance. Interestingly, TAD disruption strengthens compartmentalization, leading to a more pronounced plaid-like pattern in Hi-C maps (Figure 5C). In contrast, depleting the cohesin unloader WAPL has the opposite effect: cohesin accumulates at CTCF-binding sites, forming elongated loops, thereby reducing compartmentalization strength [185,186,187]. This indicates that cohesin-mediated looping and chromatin compartmentalization via phase separation represent competing mechanisms [188,189]. Cohesin-mediated looping also appears to compete with Polycomb-dependent interactions in mESCs, as cohesin depletion consequently strengthened long-range contacts within Polycomb chromatin domains [190]. At the single-cell level, acute cohesin removal depletion induces mixing of accessible chromatin domains, resulting in widespread gene coactivation, suggesting that cohesin prevents aberrant cross-domain chromatin clustering [191,192], in agreement with increased compartmentalization upon cohesin depletion at the cell population level [158,184]. Cohesin depletion is also reversible—upon auxin wash-off, previously lost loop domains reappear. Furthermore, the cohesin recovery rate likely depends on chromatin state, as loop domains reappeared more quickly in regions enriched with active histone marks [184] possibly due to preferential cohesin loading at accessible chromatin. Similarly to CTCF depletion, acute cohesin degradation has only a modest effect on gene expression in the short-term [164,184,193]. A few studies have provided insights into this phenomenon. First, many loops with CRE-enriched anchors (regulatory loops) persist after cohesin depletion in contrast to the loss of most structural cohesin/CTCF-mediated loops [164]. Second, cohesin has been shown to mediate transcription regulation, as its depletion promotes RNAPII release from pausing [194]. Although cohesin loss disrupts genome-wide chromatin architecture, this is counterbalanced by persistent regulatory loops and rapid transcription initiation, resulting in only mild transcriptional changes.

STAG1 and STAG2 are mutually exclusive subunits of the cohesin complex. Although they share homologous RAD21- and CTCF-interacting domains, they are distributed across overlapping and distinct genomic sites. Depletion experiments reveal distinct functional roles: STAG1-associated cohesin exhibits a longer chromatin residence time, which is enhanced by ESCO1-mediated SMC3 acetylation, which confers protection against WAPL-mediated unloading. This results in the formation of longer, more stable loops. In contrast, STAG2 loss primarily disrupts short-range interactions but moderately increases loop size at shared binding sites, likely due to compensatory recruitment of STAG1-associated cohesin [195]. Thus, STAG1- and STAG2-associated cohesin complexes contribute differently to chromatin looping, maintaining long- and short-range loops, respectively.

Cohesin cofactors have an essential role in the regulation of loading, unloading, and extrusion activity of cohesin. Cohesin cofactors interact with the limited number of sites on cohesin-STAG1/STAG2 complexes, creating competition between different cofactors. This competition defines cohesin residence time and loop extrusion rate, therefore affecting loop size and compartmentalization. NIPBL (Scc2 in yeast) in complex with MAU2 was initially proposed to act solely as a cohesin loader, but growing evidence suggests that NIPBL is also an important factor required for cohesin’s ATP-dependent extrusion activity [68,105,109,196,197,198] and in depletion experiments in live cells [199]. Thus, given NIPBL’s role in cohesin association with chromatin and extrusion activity, its depletion is expected to have a similar effect to the degradation of main cohesin subunits [118,197,199,200,201,202].

Cohesin loading and processivity are antagonized by PDS5 and WAPL [158,185,186,200]. PDS5, in complex with WAPL, facilitates cohesin unloading from DNA by competing with NIPBL for cohesin binding. This competition halts the extrusion activity of acetylated cohesin and recruits WAPL to initiate unloading. CTCF binding counteracts this process by protecting cohesin from WAPL-mediated unloading, thereby stabilizing the complex as a static loop anchor. Consequently, PDS5 depletion has a similar effect to WAPL loss, resulting in extended chromatin loops. Beyond restricting excessive loop elongation, WAPL-mediated unloading also replenishes the free cohesin pool. As a result, WAPL depletion diminishes chromatin ‘fountains’ formed by active cohesin loading at enhancers [187]. Interestingly, WAPL and NIPBL appear to counterbalance each other’s functions even in their absence, as their co-depletion unexpectedly restores the expression of most genes that are misregulated upon individual depletion [200].

Taken together, the interplay between cohesin cofactors defines a dynamic equilibrium in the formation of cohesin-mediated chromatin loops during interphase: while NIPBL promotes cohesin loading and extrusion processivity [196,197,200,203], PDS5 and WAPL facilitate unloading and recycling [158,185,186,187,200]. Targeted auxin-inducible degradation of these proteins has demonstrated how acute perturbation of any component can shift this equilibrium.

### 4.4. Transcription-Dependent Looping and Supercoiling

Genome folding mechanisms are essential for supporting tissue-specific transcription by stabilizing interactions between CREs. However, transcription itself can also shape chromatin topology through multiple mechanisms. For example, actively transcribed genes have been shown to form elongated loops [204] and promote microcompartmentalization [205]. In addition, the transcription machinery can act as a semi-permissive moving barrier, displacing extruding cohesin complexes to establish loop anchors [133,134,135]. While NIPBL enrichment at promoters was initially thought to reflect targeted recruitment to transcribed regions, it is now understood to arise from collisions between cohesin and the transcription machinery [134].

Acute RNAPII loss differentially affects CRE-anchored interactions: although enhancers and promoters share similar epigenetic features, promoter–promoter loops remain largely unaffected, whereas enhancer-anchored loops are markedly reduced [130], suggesting that distinct, yet poorly defined mechanisms may contribute to spatial communication between promoters. Beyond the loss of transcription-dependent loops, RNAPII depletion also leads to the emergence of new contacts, primarily mediated by cohesin/CTCF-dependent extrusion [128,206] and Polycomb-associated interactions [130], in line with RNAPII acting as a counterbalance to other loop-forming mechanisms. By contrast, cohesin depletion itself has minimal impact on transcription-driven loops [207].

Transcription also generates DNA supercoiling, with negative supercoils forming upstream of RNA polymerase and positive supercoils accumulating downstream, mainly enriched at transcription end sites [208]. Increased supercoiling elevates topological stress, which is resolved by topoisomerases, leading to a net increase in negative supercoiling. Notably, most type II topoisomerase inhibitors affect both TOP2A and TOP2B, making it difficult to separate their individual functions. This limitation can be addressed by using degron-tagged TOP2A or TOP2B, which allows selective depletion of each protein. Targeted depletion of type I and type II topoisomerases in interphase revealed distinct effects on supercoiling. Depletion of TOP1 or TOP2B led to increased levels of both positive and negative supercoiling at transcription start sites, transcription end sites, and domain boundaries, whereas TOP2A loss had only a minor impact, indicating a more limited role during interphase compared with other topoisomerases. Strikingly, the same study reported the presence of megabase-scale supercoiling domains (SDs) across the genome, which colocalize with chromatin compartments—negative and positive SDs aligning with A and B compartment accordingly, indicating an association between higher-order chromatin organization and supercoiling [208]. While degradation of TOP2A or TOP2B individually has only limited effects on genome architecture, loss of both isoforms increases interactions within large domains, particularly at LAD and non-LAD boundaries [138], underscoring their essential role in restricting physical crosstalk between LADs and euchromatin. At the microscopic level, topoisomerase inhibition and RNAPII depletion phenocopy each other, leading to global chromatin collapse in the nucleus due to the accumulation of positive supercoiling. Vermicelli structures that emerge in the absence of WAPL also disappear when either transcription or topoisomerase activity is impaired, indicating that cohesin function depends on the supercoiling state of DNA, as reduced negative supercoiling upon topoisomerase or RNAPII depletion may interfere with cohesin extrusion activity [131]. Interestingly, recent studies reported that cohesin, as well as other eukaryotic SMC proteins, can generate negative DNA supercoiling during loop extrusion [136,198]. Together, these observations hint at a functional interplay between topoisomerases and SMC complexes, whereby topoisomerase activity may be required for relaxation of supercoiled DNA and thereby sustain processive loop extrusion.

## 5. Current Limitations of the AID System

A major limitation of the AID system, particularly its first version (AID1), is the high level of basal degradation—protein depletion occurring even in the absence of the inducer molecule. In depletion experiments, cells carrying an AID-tagged protein but not treated with auxin are often used as a control. However, such cells may already display a hypomorphic phenotype due to reduced protein levels. In a pioneering study of targeted CTCF depletion, Nora et al. [157] reported that AID-tagged CTCF in mESCs was expressed at 2–3-fold lower levels than untagged CTCF in the parental cell line. This reduction likely reflects basal degradation, as RNA-seq data for the CTCF gene were consistent in both AID-tagged and WT cells. Consistently, a parallel study [158] found that the number of identified loops in CTCF-AID cells was more than three times lower than in wild-type cells, indicating that AID-tagged proteins may exhibit a hypomorphic phenotype. Similarly, RAD21-AID cell lines also exhibited a severe reduction in loops, and both RAD21-AID and CTCF-AID cells showed reduced insulation. Thus, the changes observed upon depletion may be insufficiently representative, as they compare an already hypomorphic phenotype with a fully depleted state. In a recent study by Yao et al. [208], the authors aimed to address these issues and performed conditional knockout experiments in synchronously developing mouse photoreceptor rods. They reported much more substantial changes in differential gene expression and chromatin accessibility compared with CTCF depletion studies. The authors proposed that this discrepancy may arise from basal degradation and the use of asynchronous cell cultures, which could introduce heterogeneity into the data. Consequently, a more systematic evaluation of CTCF and other architectural chromatin proteins using the AID1 and AID2 systems may be required. Although the AID2 system [183] and the newer variant [4] largely overcome basal degradation issues, experiments in synchronized cultures and comparisons with wild-type cells would provide deeper insights into the effects of protein depletion. As we mentioned earlier, transgenic CTCF expression driven by the Tet-On promoter does not completely rescue the WT phenotype [107,171], which should also be considered when planning an experiment or analyzing the data.

Another critical point is that the addition of the ligand itself can affect gene expression and even chromatin topology in mammalian cells. Specifically, IAA induces the expression of aryl hydrocarbon receptor (AHR) pathway genes across multiple cell lines [209,210]. Moreover, one of the upregulated AHR pathway genes was reported to reposition within the nucleus upon lamin B depletion [211], complicating the distinction between effects caused by lamin depletion and those resulting from IAA treatment. In addition, a meta-analysis of RNA-seq data from published studies using high concentrations of IAA (500 μM) revealed reduced expression of the key brain-derived neurotrophic factor, BDNF, in treated cells [209]. Previously, it was shown that exposure of pregnant rats to IAA resulted in microencephaly in the fetuses in a dose-dependent manner [212]. Therefore, high IAA concentrations may be neurotoxic and should be used with caution during neural differentiation. Use of AID2 or newer systems can reduce these issues, as the 5-Ph-IAA ligand is effective at much lower concentrations and has milder effects on gene expression [183,209]. Thus, the use of AID2 or newer systems offers greater benefits: less basal degradation and lower ligand concentration, which reduces side effects. However, regardless of the version, the AID system still requires genomic integration of both the E3 ligase adaptor and the AID tag, making the generation of AID-tagged cell lines time-consuming.

## 6. Conclusions

By providing unique advantages in speed, reversibility, tunability, and temporal precision, the AID system has proven extremely useful in dissecting chromatin dynamics through the cell cycle. The rapid and selective degradation of chromatin-associated proteins using the AID technology overcomes the limitations of traditional knockdown or knockout approaches, when cells are able to adapt to changing conditions through compensatory mechanisms. Application of the AID system in synchronized cell cultures has brought novel insights into mitotic chromosome assembly, chromatin reorganization during G1 re-entry, and maintenance of interphase chromatin.

Three-dimensional studies of mitotic chromosomes with acute depletion of condensin I, condensin II, or cohesin, alone or in combination, revealed specific features of the helically arranged chromosome scaffold, including second- and third-order diagonal bands on the Hi-C map. Further comparative analysis of Hi-C maps of mitotic cells across species, combined with knowledge of condensin and cohesin stoichiometry, would be extremely valuable for understanding the evolutionary diversity of chromosomal architectures.

A model of mitotic chromosome conformation derived from degron-based studies suggests a complex interplay between loop-extruding SMC complexes, including stalling, bypassing, and removing. Future AID-based studies targeting the other key mitotic regulators, such as TOP2A and KIF4A, will help clarify how mitotic chromosomes are organized. Particularly, whether Topo2a acts as a barrier to loop extrusion by condensins, or whether helical loop arrays are altered following acute TOP2A loss. Multiple degron systems may be easily tuned to study the combinatorial effects of simultaneous protein loss. In addition to their pivotal role in mitosis, Ki67, KIF4A, and condensin II are nuclear-localized and may thus be implicated in the organization of interphase chromatin. The AID system is particularly valuable for this group of proteins, enabling examination of acute depletion effects outside of their mitotic activities, thereby isolating their direct contribution to interphase chromatin architecture.

In recent years, AID-mediated studies have substantially expanded our understanding of interphase chromatin structure, particularly the functional roles of loop extrusion machinery and other architectural proteins in spatial genome organization. However, the molecular mechanisms underlying the establishment of promoter–enhancer interactions during mitotic exit and G1 remain poorly understood. Combined with high-resolution approaches such as Micro-C and super-resolution microscopy, targeted protein depletion offers a powerful strategy to identify factors that contribute to regulatory contacts. As rapid depletion allows precise temporal control, the AID system can also be employed to investigate the reassembly of the nucleolus during mitotic exit. Additionally, the AID system can be used to study the dynamics of X chromosome inactivation and folding throughout the cell cycle.

Future research directions should also include tissue-specific and inducible degron mouse models, enabling the study of chromatin folding during development, aging, and disease progression.

## Data Availability

No new data were created or analyzed in this study.

## Abbreviations

The following abbreviations are used in this manuscript:

AID	Auxin-inducible degradation
IAA	Indole-3-acetic acid
SMC	Structural maintenance of chromosomes
CT	Chromosome territories
TAD	Topologically associating domains
LAD	Lamina-associated domains
CRE	*Cis*-regulatory elements
mESCs	Mouse embryonic stem cells
DEGs	Differentially expressed genes
AHR	Aryl hydrocarbon receptor
